# COVID-19 underscores the important role of Clinical Ethics Committees in Africa

**DOI:** 10.1186/s12910-021-00696-2

**Published:** 2021-09-25

**Authors:** Keymanthri Moodley, Siti Mukaumbya Kabanda, Anita Kleinsmidt, Adetayo Emmanuel Obasa

**Affiliations:** Department of Medicine, Centre for Medical Ethics and Law, Faculty of Medicine and Health Sciences, Stellenbosch University, Tygerberg, South Africa

**Keywords:** Clinical ethics committees, Clinical ethics consultation services, Africa, Clinical ethics, COVID-19

## Abstract

**Background:**

The COVID-19 pandemic has magnified pre-existing challenges in healthcare in Africa. Long-standing health inequities, embedded in the continent over centuries, have been laid bare and have raised complex ethical dilemmas. While there are very few clinical ethics committees (CECs) in Africa, the demand for such services exists and has increased during the COVID-19 pandemic. The views of African healthcare professionals or bioethicists on the role of CECs in Africa have not been explored or documented previously. In this study, we aim to explore such perspectives, as well as the challenges preventing the establishment of CECs in Africa.

**Methods:**

Twenty healthcare professionals and bioethicists from Africa participated in this qualitative study that utilized in-depth semi-structured interviews with open-ended questions. Themes were identified through thematic analysis of interviews and open-ended responses.

**Results:**

Kenya and South Africa are the only countries on the continent with formal established CECs. The following themes emerged from this qualitative study: (1) Lack of formal CECs and resolution of ethical dilemmas; (2) Role of CECs during COVID-19; (3) Ethical dilemmas presented to CECs pre-COVID-19; (4) Lack of awareness of CECs; (5) Lack of qualified bioethicists or clinical ethicists; (6) Limited resources to establish CECs; (7) Creating interest in CECs and networking.

**Conclusions:**

This study illustrates the importance of clinical ethics education among African HCPs and bioethicists, more so now when COVID-19 has posed a host of clinical and ethical challenges to public and private healthcare systems. The challenges and barriers identified will inform the establishment of CECs or clinical ethics consultation services (CESs) in the region. The study results have triggered an idea for the creation of a network of African CECs.

**Supplementary Information:**

The online version contains supplementary material available at 10.1186/s12910-021-00696-2.

## Background

The coronavirus pandemic has raised numerous clinical and ethical challenges globally. Some were vaguely familiar from pre-COVID-19 times such as the fair allocation of scarce healthcare resources including beds and ventilators in intensive care units (ICUs) [1]. However, in Africa, the scarcity of all resources was exacerbated by the COVID-19 surge and this scarcity was extended to include high flow nasal oxygen (HFNO), personal protective equipment (PPE), ventilators, general hospital beds, and testing kits [2, 3]. In some instances, healthcare professionals (HCPs) struggled with the ethical conflict inherent in choosing between their lives, the health and lives of their families, and those of their patients. Many high-income countries were able to access clinical ethics committees (CECs) to assist HCPs and hospital administrators to create policies and protocols to outline clinical standards for treatment and fair distribution of scarce resources [1]. In the United States, some CECs reported that their workload doubled or increased substantially during the first 3–4 months of the pandemic [4]. This increase prompted ethics consultants to focus on providing support to HCPs who were dealing with ethical challenges surrounding critically ill patients. In addition, one of the consultants indicated that the reason they received an increase in requests for consultation was that HCPs found them useful and were satisfied with the resolution of the dilemma [4]. In South Africa, two new CECs were established during the pandemic—a COVID-19 CEC was set by the Mediclinic hospital group that has 52 health facilities in South Africa and a provincial CEC was established in the Western Cape. Some of the authors (KM, AK) served on these CECs that received several requests for consultation. Consequently, the CECs met more frequently during the pandemic. With the current third wave in South Africa, these requests are again being directed to existing and new CECs to assist with difficult triage decisions for ICU cases and hospital beds (KM-personal communication).

CECs play an integral role in clinical decision-making in hospitals and clinics as a multidisciplinary group trained in analysing and discussing clinical ethics challenges, using a systematic approach following a specific deliberation method that encourages openness about value judgments and justifications in daily clinical practice. [5, 6]. CECs play several roles in order to fulfill their functions, which include the roles of analyst, moderator, disseminator, facilitator, stakeholder advocate, advisor, and guardian of values and laws [5]. The role is well defined in high-income countries (HICs) such as in Europe, the United States, and Canada [6, 7], where CECs are regularly presented with various ethical dilemmas (end-of-life decisions, consent, confidentiality, and respecting family or surrogate wishes) [8–10]. However, according to the literature, there are no formal established CECs in Africa, other than two CECs in South Africa [11]. This raises the question of how HCPs in other African countries are handling ethical issues while caring for their patients, especially during the COVID-19 pandemic. Most HCPs in Africa have limited or no medical ethics training [12–14]. The ethics training in the region is predominantly in research ethics and Research Ethics Committees (RECs) are well established in most African countries. These RECs are predominantly responsible for the review of research protocols with the ultimate goal of protecting the rights of research participants. CECs, on the other hand, provide oversight in clinical environments such as clinics and hospitals and advise on ethical dilemmas that arise in clinical care. In the absence of CECs during the pandemic, HCPs have been forced to take responsibility for soul-wrenching decisions on triage and resource prioritization [1]. In other low- and middle-income countries (LMICs) such as Malaysia, the limited number of CECs have turned to online consultation services to provide clinical ethics advice to HCPs to help resolve the ethical dilemmas raised by the COVID-19 pandemic [15]. Such a platform could also be useful to provide support to African HCPs who have no access to clinical ethicists to provide guidance and assistance when there are conflicting values among patients, their families, and professional staff in a clinical context or CECs at their institutions.

In our previous paper [16], we explored the existence of CECs in Africa to establish baseline information and to raise awareness of the important role that CECs can play. Moodley et al. [16] found there were very few formal CECs on the continent indicating an urgent need for the establishment of CECs in Africa. Furthermore, the study found that the majority of HCPs and bioethicists surveyed were aware of ethical dilemmas in healthcare, but the concept of formal CECs was foreign as most of them were familiar with research ethics committees (RECs) only. Therefore, in this paper, we provide an in-depth insight into the perspectives of HCPs and bioethicists on CECs in Africa especially during the COVID-19 pandemic, through qualitative interviews as a follow-up study to our previous quantitative study [16].

## Methods

### Study design and sampling

In-depth interviews were used to explore participants’ views on CECs in Africa. The sample consisted of 20 African HCPs who were also bioethicists engaged in conceptual and empirical work arising from the medical and non-medical life sciences [17] or academics from the original 109-study population. All countries in Africa were invited to participate on a voluntary basis. The purposive sampling of 20 study participants from the 109-study population was concluded when data saturation occurred. We ensured that the participants selected were representatives from different parts of the African continent. Interviews were conducted from September 2019 to July 2020. Ethics approval was granted by the Faculty of Medicine and Health Sciences Health Research Ethics Committee (HREC REF: N19/05/064) at Stellenbosch University, South Africa.

### Data collection

Twenty interviews were conducted telephonically using Skype or WhatsApp calls to gather in-depth information related to CECs in Africa. As part of the consent process, participants received assurances of confidentiality and it was clarified that their participation was voluntary. Their feedback was kept anonymous by using a code number for each participant. Recordings were stored on the researcher’s computer with a password for data security purposes. Before the interview, participants were told about the aims and the objectives of this study. The interviews were audio-recorded with the consent of the participant before the interview. Questions were related to the strategies used to solve ethical problems, challenges experienced by CECs, barriers to establishing CECs, and how CECs can be established in Africa (see Additional file 1). Participants also responded to open-ended questions on the importance of CECs or consultations during the public health emergency following the COVID-19 outbreak (see Additional file 2). We recontacted all participants who were interviewed prior to the pandemic to explore their perspectives on CECs related to the public health crisis.

### Data analysis

The completed interviews were initially transcribed while maintaining anonymity and, subsequently analyzed manually by identifying the key themes. This required the researchers to familiarize themselves with the interview transcripts and was followed by organizing and categorizing responses into major themes [18]. During the analysis, two authors (KM, SMK) independently analyzed the data, compared and discussed the generated themes until consensus was reached. The trustworthiness of the data was enhanced by sharing and discussing the key themes among the study authors.

## Results

### Key themes

Qualitative analysis revealed seven themes including (1) Lack of formal CECs and resolution of ethical dilemmas; (2) Role of CECs during COVID-19; (3) Ethical dilemmas presented to CECs pre-COVID-19; (4) Lack of awareness of CECs; (5) Lack of qualified bioethicists; (6) Limited resources to establish CECs; (7) Creating interest in CECs and networking.

### Lack of formal CECs and resolution of ethical dilemmas

The majority of the participants indicated that they did not have formal CECs in their healthcare facilities, with some indicating they had research ethics committees (RECs) only. This was confirmed in the quantitative survey [16]. Respondents described how they handled clinical ethical dilemmas in general:

“We had research ethics in the teaching hospital but no clinical reference for clinical ethics…” [Country 4]

“There are no committees, but there are regulations that are set at the medical council about what they should do if there are any ethical dilemmas. Usually, the advice is that they have to sit down as experts and…have to take the decisions in consensus.” [Country 11]

“So, every now and then, I will get a call. But one thing I know hospitals do is that when they have a clinical problem they will constitute a committee… they don’t call it the ethics committee…just decide to put people together and discuss so it is an ad hoc committee” [Country 2].

Others resolved ethical dilemmas in their clinical meetings:

“…during clinical meetings, we discuss not just the usual cases but also discuss ethics-related cases.” [Country 13]

One respondent described a more informal type of ethics consultation:

“…individuals handle problems as they come. People hold conversations with each other in the corridors but there is no formal structure where you can present issues.” [Country 7]

### Role of CECs during COVID-19

When asked where HCPs were reaching out for ethical advice during the current coronavirus pandemic, those without CECs responded as follows:

“At the moment there are no proper ethical structures set to help out healthcare professionals. This is so because there are no clinical ethics committees in the hospitals in my country.

Individual healthcare workers sometimes ask bioethicists on a personal level but not as institutional arrangements.” [Country 12]

“Healthcare professionals are reaching out for ethical advice during the current major global health emergency based on the experience of the senior colleagues/consultants who are working with the frontline teams.” [Country 13]

“For my country, there is no system for answering ethical questions in hospitals, no committees for professional ethics, and this affects the direct answer to doctors' ethical concerns. Most doctors turn to the Internet and ask their older colleagues or the more experienced doctors”. [Country 16]

The two countries that indicated they had CECs received support from their local committees during the pandemic, as described below by one participant:

“A provincial CEC was established in one province specifically for COVID-19 dilemmas and a private hospital group also established a COVID-19 CEC at the start of the pandemic” [Country 1].

“At the National Referral Hospital, the Clinical Ethics Committee is being consulted and is guiding on ethical issues during the pandemic. This is also happening at the University Hospital”. [ Country 6]

Most participants indicated that having a CEC within healthcare facilities was important, as it would help with complex decision-making and would support the staff working on the frontline during COVID-19 who have to make difficult decisions*.* Furthermore, the participants felt having a CEC would have prepared HCPs in handling ethical dilemmas due to the COVID-19 pandemic.

“The CEC would be important in guiding HCPs and especially in this time of a challenging new virus. Everyday HCPs are dealing with new dilemmas when providing care to COVID-19 patients, so the CEC would help by handling that burden and making sound ethical decisions [Country 12]

“CECs would have established policies that address many of the common and uncommon ethical issues. These policies would have been helpful during the pandemic. It is easier to establish policies to deal with emergencies during non-emergency periods as it allows non-emotional deliberations and allows adequate time for discussion and exploration of different viewpoints and involves more stakeholders. Policies to address issues such as allocation of scarce resources, do not resuscitate orders, quarantine, etc…” [Country 6]

“We think that having clinical ethics committees would prepare healthcare professionals for this global health emergency. They encounter different ethical aspects: lack of materials, treatment protocols, refusal of treatment from the patient.” [Country 8]

“CECs are important because it will help institutions make well informed clinical decisions especially for clinically ill patients like those suffering from COVID-19 where clinicians in ICU need to decide which patient to put on a ventilator and which ones not to...” [Country 5]

“…because CEC will provide a platform for clinicians and other health care workers, to obtain needed help in deciding what to do when faced with an ethical-legal challenge in practice…”[Country 13]

Another participant interestingly mentioned how the pandemic ignited the intention to establish a CEC.

“Until today, there was no intent to do it but the situation such as the Covid-19 pandemic heightened the urgent need to put it in place”. [Country 8]

During the current COVID-19 pandemic, participants had several ethical issues or dilemmas that they were confronted with within their countries. Most of these issues involved resource allocation, patient and staff safety.

“…At our institution, we are facing issues of allocation of resources such as ICU beds, ventilators, High Flow Nasal Oxygen and PPE. There was also conflict over de-escalation of surgical services to deploy skilled nursing staff to high care and critical care units. One case that was particularly difficult involved a patient who was managed in another country and was repatriated with complications. His condition was futile and discussion arose around allowing his wife to see him during lockdown regulations prior to withdrawing ventilation as she had not seen him for several months while he was abroad” [Country 1]

“Most ethical decisions relate to dealing with the distribution of limited resources in a fair way to patients. In addition to that, the ethical dilemma of medical staff in danger of dying while carrying out their professional duties became important”. [Country 16]

“… there is a resource constraint on COVID-19 such as lack of PPE and other equipment like ventilators. The issue was who gets to be put on a ventilator in case of need for respiratory support…” [Country 12]

### Ethical dilemmas presented to CECs pre-COVID-19

Participants were asked to describe the kind of ethical dilemmas referred to in their CECs. The participants mentioned a wide variety of ethical issues:

“Beginning of life issues, end of life issues, futility…when should we stop the next round of anticancer drugs? Because sometimes the burden of treatment becomes worse than not living. Clinical problems, what is living, what is quality of living, and what is the quality of dying...Then we have a lot of problems with informed consent. Can this person give informed consent? When can children give consent? Our Children’s Act is now pushed down to the age of 12 to give consent to medical treatment. But the next line in the Act is important - the clinician must make sure that this child is of sufficient maturity and competent to make their decisions…there are also ethical problems, not only on competency but also on confidentiality. When can we break confidentiality…that at times is unclear… those are things we see mostly” [Country 1]

“The other one has been the issue on next of kin, because when there is a disagreement between relatives on the management of care of the patient, then who talks on behalf of the patient. That is a common one. Because of the nature of our societies often, the wife of the sick person may not be the one who is the spokesperson of the family even though legally the husband is the one who she is related to but it is more complicated. We also had issues of request…for sex exchange operations because the situation in our system is a bit complicated from a legal and social point of view…” [Country 6]

### Lack of awareness of CECs

The majority of the participants described a lack of awareness of CECs as one of the challenges in establishing committees in African healthcare facilities. This illustrated that not so many of HCPs or bioethicists are aware of the CEC concept:

“Many of us are unaware of the concept of CECs…when we were trying to set up the hospital ethics committee, they didn’t know the concept, they felt it was a research ethics committee Another perception is that ethics committees are those committees looking for faults or deal with doctors who have been accused of malpractice…so it will take a long time before a hospital CEC is accepted.” [Country 6]

“The thing is that people have no clue what it is, that is the major point. They know about research …I wanted to join one in the school of medicine…and the dean told me he had no idea what bioethics or clinical ethics is and this is someone who is an HCP and I have been consulting for them… to say that I was surprised is an understatement. I don’t want to use the word shocked. But then talking to other people I realised they have no clue until maybe they have serious issues and someone says this is an ethical dilemma so how should we deal with it…So it’s about lack of awareness.” [Country 2]

“… we are facing a lack of information. People need to be informed about CECs”. [Country 20]

### Lack of qualified bioethicists or clinical ethicists

Some African countries still lack expertise in bioethics or clinical ethics, which is important in the decision-making process during CESs. The participants indicated that the lack of qualified bioethicists is one of the challenges in establishing a CEC as described below:

“There are not enough bioethicists…there are not enough qualified bioethicists.”. [Country 1]

“I think generally it would be, from my understanding, a lack of expertise because the CEC is there to guide healthcare professionals to better medical care access.” [Country 9]

### Limited resources to establish CECs

Some participants mentioned a lack of resources such as access to training, financial and human resources, as some of the challenges in establishing CECs.

“so, access to training in bioethics is limited. We do not even have programs in bioethics... I know of a program that is mostly looking at research but not issues related to clinical ethics. Because the funding is NIH and the NIH interest is research, not clinical ethics. I think if we are going to see more clinical ethics, we need to dedicate funds to do clinical ethics.” [Country 7]

“resources are another issue because if we need to have CECs we need to have a small setup and someone to help us to run the office/setup, so this is quite costly. It is about providing training that will lead to the establishment of CECs within the hospital setting. It can be replicated in other big hospitals in other parts of the country.” [Country 3]

### Creating interest in CECs and networking

Inculcating interest in the development of CECs was also among the challenges mentioned in establishing such committees.

“I think the first thing is the will; you must employ someone or a few doctors who are interested in this topic and put them together. So the first step is usually difficult. Of course, for interest, the subject is very important.” [Country 8]

“I think one of the major challenges is the will or initiative. Because we do not have the initiative to start the CEC…something has to be started by someone. We do not have initiative for that …the bigger issue is to employ people to be part of the committee…” [Country 5]

Some of the participants indicated that raising awareness about CECs among HCPs could be a start in contributing to establishing a CEC in their healthcare facilities.

“One needs to create awareness with our doctors or healthcare professionals. When you create the awareness then the need for it comes spontaneously”. [Country 6]

“…I think we should put some effort into informing health professionals about the importance of such committees…making it known what the job is all about and why this committee is important in hospitals, from my country I think is the problem.” [Country 11]

Other participants suggested that channels of educating and training the professionals could contribute to establishing CECs in their regions.

“Education, Education, Education so have seminars, have workshops, write academic papers, reach out to conferences.” [Country 2]

“…many people need training because we hear about CECs, but we don’t know what it involves, what it includes.” [Country 18]

While other participants suggested the importance of networking and collaborating with other established CECs.

“I think we start first with establishing a network for CECs and then we can identify the focal person in each African country who wants to be involved in that…then identify the gaps. If it is training, then we have to look at African countries that have well-established CECs so they can transfer their experiences to other countries that do not have…So I think having a platform for African countries interested in establishing such committees will be helpful, in exchange for ideas and expertise.” [Country 11]

## Discussion

Our study provides insight into HCPs/bioethicists' awareness of and perspectives on CECs in Africa. According to our knowledge, this is the first published study describing the experiences and perspectives of professionals on CECs in Africa. This qualitative study has confirmed that most of the participants lacked formal CECs in their institutions and countries, as seen in our previous quantitative paper [16]. Only two African countries, South Africa and Kenya mentioned having established CECs with multidisciplinary membership that has been operating for more than three years. However, participants from other African countries without formal CECs described alternative approaches that they used in resolving ethical dilemmas. These included ad hoc consultations, clinical meetings, using existing knowledge, or seeking advice from colleagues. These approaches have been observed in other resources poor and some resource rich countries as well [13, 19–22]. However, in a morally pluralistic world, it is insufficient to depend on individual professional judgment and moral codes to provide high quality healthcare because the quality of ethical deliberation required to make good healthcare decisions requires diverse clinical and ethical expertise [21]. This expertise can be acquired by having a multidisciplinary CEC with members who are trained or experienced in applying ethics frameworks to aid decision-making with respect to clinical dilemmas.

In the midst of the coronavirus pandemic, participants realised the importance of having CECs in African healthcare institutions as this would have better prepared the health workforce for decision-making during this public health crisis. There were new ethical issues involving clinical decisions in critical care units, allocation of limited resources, and treatment of surgical patients. The support of CECs has the potential to provide valuable support to HCPs and healthcare institutions enabling decisions based on sound judgment, concrete understanding of ethical principles, and the ability to engage with patients with transparency. In particular, ethical challenges arose in the allocation of scarce resources and the prioritisation of treatment and care for patients with co-morbidities in under-resourced African countries [23]. Non-COVID-19 health services (HIV, Tuberculosis, oncology, and non-communicable diseases) were de-escalated to prioritise acute COVID-19 care. This created moral distress for HCPs managing non-COVID-19 wards and clinics. Furthermore, many HCPs on the frontlines have reported experiencing moral distress and moral injury [24]. Moral distress usually arises when HCPs are unable to carry out their tasks or when they are forced to deny essential and life-saving treatment to a patient due to a lack of resources. Consequently, this level of distress may lead to feelings of anger, shame, and guilt, which if left unresolved may cause moral injury that may lead to anxiety and depression [25]. CECs play an important role in creating distance between the dilemma and the HCP minimising moral distress and moral injury in HCPs who are expected to deliver care under extremely difficult conditions [26]. It is therefore unsurprising that some of the CECs in the USA noted that their ethics consultations doubled during the pandemic [4], and in certain facilities, they had to educate staff by implementing a series of formal and informal education programs at the bedside and via virtual ethics consultation services. Such online ethics consultation services could provide workable alternatives to physical on-site CECs, as implemented in Malaysia [15], especially during the current coronavirus pandemic. The online platform can be coordinated by clinical ethicists in the country or outside the country, where HCPs ask their questions or gain access to resources related to clinical ethics concerns to guide their decision-making processes. Such online platforms have worked well for RECs during the pandemic and could work equally well for CECs provided sufficient security measures are in place to preserve confidentiality.

Several legal concerns arose during the pandemic [27]. Although our findings show that African countries with no formal CECs are receiving support from individual ethicists formally or informally, having formal ethics support services such as CECs could enhance the clarity, consistency, and plausibility of decisions made across African countries [28]. This is especially necessary from a legal perspective. For example, in Nigeria, there has been increasing litigation against HCPs, which might indicate the gaps between their level of awareness and basic knowledge of medical ethics [12]. South Africa has been experiencing an increase in medical litigation for the past decade [29, 30] because patients have become more aware of their legal rights. This could increase after the pandemic but it is currently too early to assess this possibility. In South Africa, our institutional CEC has been assisting with mediation when conflict arises between the healthcare team and families of patients since 2009. The mediation role of CECs could potentially be used to reduce medico-legal issues and conflicts to simplify a pragmatic reconciliation that allows a path forward in clinical care [30].

While ethical dilemmas increased substantially during the pandemic, respondents indicated that formal CECs in Kenya and South Africa were presented with many complex ethical dilemmas pre-COVID-19 as well. These dilemmas included consent difficulties, end-of-life decision-making, confidentiality, and conflicts regarding surrogate decision-making. Similar dilemmas have also been observed in developed countries [8, 9, 10, 32–34]. One of the study participants indicated that their CEC received referrals related to consent, particularly regarding the child’s capacity to provide informed consent. Such situations can be complex as parents in Africa often believe they know what is best for their children [35]. In a Norwegian study, the authors found that the “more people have a say in medical issues, conflicts regarding solutions of complex medical problems obviously become more frequent”. This could explain why 19 of their 31 case studies involved children who had appointed their guardians (parents) to make medical decisions [36]. In these situations, CECs or CESs balance all the possible treatment options before concluding what would be in the best interests of the minor [37]. However, an additional challenge is trying to respect patient autonomy with respect to the competence of children [37]. With such challenges, the competence of children to make various decisions about different aspects of their health care must be viewed on a sliding scale [37].

Another complicated ethical dilemma handled by an African CEC is related to end-of-life decisions. This has also been observed in another study, where Norwegian doctors brought end-of-life decision cases to their CEC [31]. It was found that half of the cases involved conflicts with relatives of the patients. In most of the cases, there was uncertainty about what would be the ethically preferable solution. In such instances, a clinical ethics consultation was the solution for HCPs to get broader illumination or moral backing on a decision being contemplated by the medical team.

Dilemmas around confidentiality are also presented at the CEC. One participant mentioned that some HCPs are not sure when confidentiality can be breached or preserved. Although confidentiality is one of the important moral values in patient care, open communication is sometimes necessary but on the other hand, there is a strict obligation to confidentiality with certain narrow exceptions. In a bid to help in times of serious conflict, CECs or CESs greatly depend upon the trust of all members involved, in the knowledge that patients’ personal rights will be respected [38].

Sometimes, the nature of the ethical dilemmas can be complicated as issues of communication, information, family conflict, and futility give rise to ethical quandaries [32, 36, 39]. Nevertheless, these ethical dilemmas and others may highlight the need for clinical ethics education in healthcare facilities to train HCPs to handle them, even where CECs do not exist.

A lack of awareness of CECs and expertise in clinical ethics were among the major challenges identified in establishing CECs. To a large extent, the awareness challenge could be due to the fact that unlike RECs or Institutional Review Boards (IRBs), ‘CEC’ is a new concept in most resource-poor countries. This finding illustrates the importance of educating and training CEC members to execute their functions efficiently and effectively to build credibility within healthcare institutions [40]. Funding constraints in establishing CECs are related to the emphasis on RECs or IRBs in Africa. Most research funders like the National Institutes of Health (NIH) and European and Developing Countries Clinical Trials Partnership (EDCTP) fund development of research ethicists, not bioethicists and clinical ethicists and they fund the establishment of RECs or IRBs, not CECs. Furthermore, the lack of ethical guidance or expertise is compounded during a pandemic, as smaller and weaker health systems are at greater risk of using all their resources for healthcare with little or no support from the government for ethics [41].

Lastly, the lack of interest in setting up such committees was related to a lack of awareness of CECs. While conferences focusing on research ethics are commonplace in Africa, there are very few events focusing on clinical ethics. The International Conference on Clinical Ethics and Consultation (ICCEC) has been hosted globally for the past 15 years, but never in Africa. Very few African delegates have been able to attend this conference when it was hosted in the global north. However, in 2021, ICCEC will be hosted in South Africa for the first time in its 15-year history (www.iccec2021.co.za). This will provide an important role in stimulating interest in clinical ethics on the continent.

### Study limitations

Our study has many strengths but also a few limitations. By interviewing bioethicists/HCPs, we were able to obtain their unique experiences and perspectives on CECs in Africa, which is a major strength. In addition, through in-depth interviews, we were able to clarify the differences in understanding between RECs and CECs to some of the participants that were not so familiar with the term “CEC”. A limitation was the suboptimal response from Lusophone and Francophone African countries. Most of the interviews were conducted in English as we did not have French- or Portuguese-speaking research assistants in this unfunded project. Although we could not interview participants from all African countries, we managed to interview representatives from many different parts of the African continent including some Francophone and Lusophone countries where participants were able to communicate in English. Given the suboptimal response from Lusophone and Francophone countries, future studies could attempt to source funding and translators to ensure balanced responses from all parts of Africa to improve the representativeness of the data. We validated our findings by analysing and discussing the data with the research team. Further research should investigate African countries that have implemented CECs in their healthcare facilities since this study was conducted or as a result of the coronavirus pandemic. It would also be interesting to examine whether CECs have an impact on the delivery of healthcare in African countries and the impact on healthcare litigation in Africa.

## Conclusion

Our study has assisted in promoting awareness of CECs and recognition of the importance of establishing CECs in many African countries. Specialised training for HCPs in the area of clinical ethics was identified as a critical need amongst respondents in this study. Engaging with participants in Francophone and Lusophone countries in Africa would add to the diversity of perspectives in future studies. The timing of this study was pivotal in creating an appreciation of the need for CECs during the coronavirus pandemic. New CECs were developed and maintained during the first and second waves of infection in South Africa and are to be retained after the pandemic due to the positive role played. The idea for the formation of a network of CECs in Africa has been developed during this study and we hope to implement this finding and launch the first CEC network in Africa at the International Conference on Clinical Ethics and Consultation (ICCEC) in South Africa in 2021. The overall aim of our proposed CEC network is to develop and sustain multidisciplinary expertise in clinical ethics in Africa. The objectives of the network will be to provide capacity building to developing and established CECs, organise annual meetings, establish contacts with CECs internationally, produce a database of information for CECs, and encourage resource sharing across African countries.

## Supplementary Information

**Additional file 1**: Interview guide for Promoting the establishment of Clinical Ethics Committees (CECs) in Africa.

**Additional file 2**: Interview guide for Importance of clinical ethics committees or consultations during public health emergency over the COVID-19 outbreak.

## Data Availability

Anonymised datasets used during the current study are available from the corresponding author.

## Abbreviations

CEC: Clinical ethics committee
CESs: Clinical ethics consultations services
HCPs: Healthcare professionals
HFNO: High flow nasal oxygen
HICs: High-income countries
HRECs: Health research ethics committees
ICCEC 2021: International Conference on Clinical Ethics and Consultation 2021
ICU: Intensive Care Units
IRB: Institutional review board
LMICs: Low- and middle-income countries
PPE: Personal Protective Equipment
RECs: Research Ethics Committees
