# Supplementary material for: COVID-19 underscores the important role of Clinical Ethics Committees in Africa
Source: BMC Med Ethics. 2021 Sep 25;22:131. doi: 10.1186/s12910-021-00696-2 (PMC8465788; doi:10.1186/s12910-021-00696-2)
Supplement: Supplementary file 1 — Additional file 1: Interview guide for Promoting the establishment of Clinical Ethics Committees (CECs) in Africa. [file 12910_2021_696_MOESM1_ESM.docx]

**Interview Guide: Promoting the establishment of Clinical Ethics Committees (CECs) in Africa**

Healthcare in the 21st century is fraught with ethical dilemmas. Advances in life-prolonging technologies have created dilemmas that are important, value-laden, and highly complex. With the increasing awareness surrounding the ethical dimensions of clinical practice, Clinical Ethics Committees (CECs) are established in order to provide services that ensure high standards of ethical practice. The term ‘clinical ethics’ is still under-utilised in many developing countries. In some regions in Africa, many people lack adequate resources, access to healthcare professionals, and reasonable healthcare. As a result, focusing on ‘clinical ethics’ is challenging even though there is an especially strong need for CECs in resource-constrained environments.

**Questions**

If there is an established CEC in your country,

o What are the details thereof?

o What is the professional status of committee members?

o Does the committee have administrative support? (part-time/full-time)

o What challenges do you as a committee face?

o What are the clinical ethics issues that your committee deals with?

o Have committee members received training in ethics?

If there is no established CEC in your country,

o How are clinical ethics issues dealt with?

o Who are the professionals that deal with clinical ethics issues?

o What challenges do you face from establishing a CEC in your region?

Improvement of CECs in Africa

o Are you aware of CECs in Africa? And if so, please provide details

o Are there any healthcare professionals/ethicists that you know that are involved in CECs?

o What challenges do African countries face w.r.t establishing a CEC?

o How can healthcare professionals/ethicists contribute to establishing a CEC in their region?

ICCEC 2021

o Would you say that there is a need for a workshop that focuses on CECs in Africa? Why?

o Would you be interested in attending a workshop part of the ICCEC conference that focuses on building CECs in Africa?

o Would you consider such a space appropriate for skills development and capacity building?

o Are you aware of the Hans Joachim Schwager Award for Clinical Ethics that rewards building clinical ethics capacity?

Is there anything else that you would like to contribute or feel that I have missed anything?

Thank you for your participation in this interview
