# Supplementary material for: COVID-19 underscores the important role of Clinical Ethics Committees in Africa
Source: BMC Med Ethics. 2021 Sep 25;22:131. doi: 10.1186/s12910-021-00696-2 (PMC8465788; doi:10.1186/s12910-021-00696-2)
Supplement: Supplementary file 2 — Additional file 2: Interview guide for Importance of clinical ethics committees or consultations during public health emergency over the COVID-19 outbreak. [file 12910_2021_696_MOESM2_ESM.docx]

**Interview Guide: Importance of clinical ethics committees or consultations during public health emergency over the COVID-19 outbreak.**

**Questions**

We wanted to follow-up with few questions regarding the importance of clinical ethics committees or consultations during this public health emergency over the COVID-19 outbreak.

o With the current situation of COVID-19 that we are facing, what are the ethically complex decisions that healthcare professionals are faced with within your institution or country? And how are they handled?

o Do you know or have an idea of who or where healthcare professionals are reaching out for ethical advice during the current major global health emergency?

o With the COVID-19 pandemic, does your institution or organizations/institutions elsewhere in your country intend to create a clinical ethics committee or emergency clinical ethics consultation?

o Do you think that having clinical ethics committees or consultation services in your institution or country would prepare healthcare professionals for this global health emergency? If yes, how? And If no, why?
